# Assessing the Patient Outcomes and Performance of a Cardiothoracic and Vascular Surgery (CTVS) Unit During Its First Two Years in a Tier-2 City in India: A Comprehensive Audit and Analysis

**DOI:** 10.7759/cureus.42910

**Published:** 2023-08-03

**Authors:** Anuj Tiwari, Abhishek Sharma, Sofia Jaswal, Suzen s Kaur, Niketa Thakur

**Affiliations:** 1 Department of Surgery, Sri Guru Ram Das (SGRD) Institute of Medical Sciences and Research, Amritsar, IND; 2 Department of Anaesthesia, Sri Guru Ram Das (SGRD) Institute of Medical Sciences and Research, Amritsar, IND; 3 Department of Anesthesia and Critical Care, Homi Bhabha Cancer Hospital and Research Center, Chandigarh, IND; 4 Department of Anesthesia, Sri Guru Ram Das (SGRD) Institute of Medical Sciences and Research, Amritsar, IND; 5 Department of Radiation Oncology, Government Medical College, Amritsar, Amritsar, IND

**Keywords:** coronary artery bypass grafting (cabg), cardiac valvular surgery, off-pump cabg, post-operative outcome in cardiac surgery, fast tracking, antimicrobial resistance, low cardiac output syndrome, intra-aortic balloon pump (iabp), drug abuse, cardiothoracic and vascular surgery

## Abstract

This detailed article presents a comprehensive overview of the initial two-year experience in establishing a new cardiothoracic vascular surgery (CTVS) facility in a tier-2 city in India. The article discusses various aspects of setting up and operating a specialized healthcare facility. The first two years of developing the CTVS facility were included in the study period. The manpower included one cardiothoracic vascular surgeon, one cardiac anesthesiologist, two perfusionists, and two physician assistants, along with four other ancillary staff to assist in the smooth functioning of the operation theater. The CTVS recovery staff included 15 nursing officers. There was only one modular operation theater reserved for cardiothoracic vascular surgeries, along with a five-bed recovery room (CTVS intensive care unit). One-hundred-seventy-two procedures were done, including 122 open heart surgeries, 36 vascular procedures, and 14 thoracic procedures. The majority of patients were discharged by the seventh day postoperatively. Overall complication and mortality rates were 8% and 4.6%, respectively. This article also discusses relevant hospital policy, challenges faced, and future recommendations for similar endeavors. The findings highlight the successful implementation of the facility and its impact on providing specialized cardiac care to the local population.

## Introduction

Over the last quarter-century, there has been an increasing trend of cardiovascular disease, highlighting that cardiovascular diseases are the largest contributor to the disease burden of any disease group and are a major public health problem leading to premature deaths and morbidity across all states of India, particularly in this region [1].

This trend emphasizes the urgent need for specialized cardiothoracic surgery facilities for effective disease management. In the past, the concentration of these specialized facilities has been notably high in metropolitan and tier-1 cities across India. This geographical bias in healthcare infrastructure has culminated in a considerable disparity in access to advanced healthcare services between urban and rural or semi-urban populations. In response to this disparity, concerted efforts have been made to decentralize healthcare facilities. By introducing specialized services like cardiothoracic surgery centers in tier-2 cities, we strive to bring advanced healthcare closer to semi-urban populations. This step marks a significant stride toward democratizing healthcare access across the country [2].

Despite the clear need for such decentralization, establishing a cardiothoracic surgery facility in a tier-2 city is no small feat. It entails navigating a unique set of challenges that range from infrastructural and logistical hurdles to ensuring the availability of adequately trained personnel. Additionally, overcoming socio-economic factors that may influence the acceptance and utilization of such services in these areas is equally critical.

This article delves into the journey of setting up a cardiothoracic surgery facility in a tier-2 city, highlighting the lessons learned from the challenges faced and the victories celebrated. It underlines the strides made toward bridging the rural-urban divide in specialized healthcare access in India. By shining a spotlight on this journey, we aim to inspire further efforts towards equitable healthcare delivery across the nation, truly embodying the ethos of building dreams and saving lives.

## Materials and methods

This study provides a retrospective audit and analysis of the first two years of operation (from July 1, 2021, to June 30, 2023) at our newly established cardiothoracic surgery facility, the Sri Guru Ram Dass Institute of Medical Sciences and Research in Amritsar.

Relevant patient data were obtained from patient records for this retrospective study. Key aspects of our data collection involved surgical volume, patient demographics, co-morbidities, procedures performed, and perioperative outcomes. We gathered detailed information on clinical diagnoses, biventricular function, and pulmonary arterial hypertension, along with the antibiotic protocols followed and fast-track policies adopted for each patient.

We organized patient data into three main categories: open heart surgery, thoracic procedures, and vascular procedures. Patients were further categorized according to gender and year of surgery. Patient co-morbidities were also recorded in each surgery category. We also recorded intra-operative and postoperative events such as emergency conversion to on-pump, new-onset atrial fibrillation, and postoperative low cardiac output syndrome. To measure patient outcomes, we considered metrics like mortality rates, length of hospital stay, and post-operative complications. The timelines for postoperative mobilization, ICU stay, and overall hospital stay for each patient were also noted. Additionally, data regarding substance abuse, including intravenous drugs and opioid misuse, were collected.

We employed Microsoft Excel 2010 (Microsoft Corporation, Redmond, WA) for data analysis, reporting results as mean values with standard deviations or as percentages, depending on the type of data. Patients who received only medical care or whose operations were postponed were excluded from these statistics.

This study, approved by the Institutional Ethics Committee (SGRD/IEC/2023-198), aims to offer comprehensive insights into the functioning of the new cardiothoracic surgery unit, contributing significantly to understanding how such facilities can be effectively set up and run. Informed consent was omitted due to the lack of patient contact in the present study.

## Results

Patient demographics

During the initial two-year period, a total of 172 cardiothoracic and vascular procedures were performed at the facility. The patient population consisted of adults (age > 18 years), including adult patients operated on for congenital heart diseases. There were no procedures done in the pediatric or adolescent population. The patient population comprised 121 (70%) males and 51 (30%) females, with a mean age of 53.7 ± 11.6 years for males and 66.7 ± 12.8 years for females. The overall mean age of patients at the time of operation was 57.5 ± 8.3 years. Patient demographics and mean age have been detailed in Table 1. We have also documented the associated co-morbidity and history of substance abuse in patients in Table 2.

**Table 1:** Patient Demographics and Yearly Distribution of Cases CABG; coronary artery bypass grafting, MVR; mitral valve replacement, AVR; aortic valve replacement, DVR; double valve replacement, ASD; atrial septal defect, TOF; tetralogy of Fallot, TAPVC; total anomalous pulmonary venous connections

Gender Distribution (Mean age in years)	2021		2022		2023		TOTAL
Surgery	Male	Female	Male	Female	Male	Female	
CABG	-	-	19 (63)	8 (64)	17 (55)	7 (62)	51 (60)
MVR	-	-	7 (35)	6 (48)	12 (48)	7 (58)	32 (47)
AVR	-	-	3 (51)	-	11 (54)	3 (61)	17 (54.7)
DVR	-	-	3 (49)	-	4 (35)	-	7 (41)
MVR+CABG	-	-	2 (61)	-	-	-	2 (61)
AVR+CABG	-	-	-	-	1 (65)	2 (72)	3 (69.7)
ASD	-	-	2 (57)	-	2 (25)	1 (38)	5 (40)
TOF Repair	-	-	1 (33)	1 (28)	-	-	2 (30.5)
TAPVC Repair	-	-	1 (18)	-	-	-	1 (18)
Left Atrial Myxoma	-	-	-	1 (38)	-	1 (61)	2 (49.5)
Schwannoma Removal – Mediastinum	-	1 (35)	1 (40)	-	-	-	2 (37.5)
Decortication Lung	-	1 (72)	1 (55)	3 (58)	1 (47)	-	6 (58)
Foreign Body – Thoracic Region			4 (69)		2 (56)		6 (64.7)
Axillofemoral – Femoral Bypass Grafting	-		1 (65)		-		1 (65)
Femoro- Popliteal Bypass Grafting	-	-	1 (57)	-	1 (64)	-	2 (60.5)
Artery Repair	1 (41)	-	4 (42)	3 (36)	-		7 (39)
Embolectomy	2 (54)	1 (52)	5 (50)	1 (60)	1 (38)	4 (58)	14 (53)
Pseudoaneurysm Repair – Tibial Artery Repair	-	-	1 (37)	1 (56)			2 (46.5)
Arterio Venous Fistula Formation	-	-	4 (55)	3 (47)	3 (48)	2 (52)	12 (50.7)

**Table 2:** Patient Co-morbidities in Open Heart Surgery, Thoracic Surgery, and Vascular Surgery Cases CAD: coronary artery disease, RHD; rheumatic heart disease, ASD; atrial septal defect, TOF; tetralogy of Fallot, LV; left ventricular, PAH; pulmonary artery hypertension, HTN; hypertension, COPD; chronic obstructive pulmonary disease

Clinical Condition	LV Dysfunction - Mild To Moderate (Ef-30- 50%) N(%)	LV Dysfunction - Severe (Ef<30%) N(%)	PAH (Mean Pap>25mmhg)	Diabetes Mellitus (Type-2)	HTN	Obesity	Thyroid Disorder	COPD	Chronic Kidney Disease	Opioid Abuse	Intravenous Drug Abuse
CAD	24 (47%)	2 (3.9%)	6 (11.7%)	22 (43%)	16 (31%)	12 (24%)	4 (7.8%)	4 (7.8%)	2 (3.9%)	12 (23.5%)	-
RHD	16 (30%)	4 (7.5%)	12 (22.6%)	8 (15%)	10 (19%)	6 (11%)	6 (11%)	2 (4%)	3 (5.7%)	8 (15%)	-
ASD	1 (20%)	-	1 (20%)	-	-	-	1 (20%)	-	-	-	-
ToF	-	-	-	-	-	-	--	-	-	-	-
Left Atrial Myxoma	-	-	2 (100%)	-	-	-	-	-	-	-	-
Decortication Lung	-	-	1 (17%)	-	-	-	-	-	-	-	-
Schwanomma	-	-	-	-	1 (50%)	-	-	-	-	-	-
Foreign Body –Thoracic Region	-	-	-	2 (33%)	1 (17%)	-	-	-	--	1 (17%)	-
Axillo-Femoral Bypass Grafting	-	-	-	-	1 (100%)	-	-	1 (100%)	-	-	-
Femoro- Popliteal Bypass Grafting	-	-	-	1 (50%)	1 (50%)	-	-	-	-	1 (50%)	-
Femoral Artery Repair	1 (20%)	-	-	-	-	-	2 (40%)	-	-	-	3 (60%)
Tibial Artery Repair	-	-	-	-	-	-	-	-	-	-	1 (50%)
AV Fistula Formation	-	-	2 (17%)	8 (67%)	12 (100%)	-	-	-	12 (100%)	4 (33%)	-

Distribution by clinical diagnosis

The three most common clinical diagnoses were rheumatic heart disease (RHD) in 53 cases (30%), coronary artery disease in 51 cases (29%), and acute vascular thrombosis leading to acute limb ischemia in 14 cases (8%). Significant gender differences (p value < 0.5) were noted in rheumatic heart disease (male 66% and female 34%) and coronary artery disease (male 70%, female 30%) in our study.

Distribution by operative categories and procedures

Open heart surgery (OHS) was done in 122 (71%), vascular procedures in 36 (21%), and 14 cases (8%) of thoracic surgery were done in a two-year period. (Table 2). The overall distribution of OHS procedures was coronary artery bypass surgery: 51 (42%), mitral valve replacement: 32 (26%), aortic valve replacement: 17 (14%), double valve replacement (mitral valve + aortic valve): seven (6%), aortic valve replacement + coronary artery bypass surgery: three (2.5%), mitral valve replacement + coronary artery bypass grafting: two (1.6%), atrial septal defect closure: five (4%), tetralogy of Fallot (ToF) repair (intra-cardiac repair): two (1.6%), left atrial myxoma removal: 1.2%, mediastinal schwannoma removal: 1.2%, total anomalous pulmonary venous connections repair: 0.6%, foreign body removal in the thoracic region: 3.5%; and decortication right lung: 3.5% (Table 2). The overall mortality registered was eight (4.6%).

Open heart surgery (OHS) procedures

We initially started with thoracic and vascular surgery in July 2021 while OHS was introduced in January 2022, and till now, a total of 122 cases of open heart surgery have been performed. The most common procedure done was related to rheumatic heart disease (RHD). A total of 54 cases were done for RHD sequelae, which included mitral valve replacement surgery (60% of total valve replacement procedures) and aortic valve replacement procedures (28% of total valve replacements). A total of three and five cases were done for calcific aortic valves and bicuspid aortic valves respectively. Among valve replacement surgeries, a total of 21 cases were done in the year 2022, while 40 cases were done in 2023.

Among 51 cases of CABG, 70% were males with a mean age of 59 years, and 30% were female patients with a mean age of 63.6 years. A total of 27 and 24 cases of CABG were performed in 2022 and 2023, respectively. The majority of CABG was done off pump: 36 (70%). In 11 (21%) cases, the procedure was electively done on cardiopulmonary bypass while in four (8%) cases, an emergency cardiopulmonary bypass was instituted. The average ICU and hospital stays were 2.3 ± 1.1 days and 6 ± 1.8 days, respectively. We also performed procedures on adult patients with congenital heart disease, including intracardiac repair for TOF, total anomalous pulmonary venous connections (TAPVC) repair for total anomalous pulmonary venous connections, and surgical ASD closure for an atrial septal defect with an uneventful postoperative period.

Thoracic Procedures:

A total of 14 thoracic procedures were performed: six patients (43%) were operated on for decortication for chronic empyema (four for left and two for right lung), six patients (43%) had foreign body removal done from the thoracic region, and two patients (14%) had schwannoma removal from the posterior mediastinum. There were 64% male and 36% female by gender distribution in thoracic cases, with a mean age of 58 ± 12 years in males and 56 ± 10 years in females. There was no mortality recorded in thoracic procedures. While average ICU and hospital stays were 1.8 ± 1.4 days and 7 ± 1.2 days, respectively.

Vascular procedures

We performed 36 vascular procedures, with thromboembolectomy in 14 patients (39%) for acute arterial thrombosis and arterial venous fistula formation in 12 patients (34%) with chronic kidney disease (CKD) being the most frequent. The femoral artery was the most commonly repaired vessel, involving five patients (14%), followed by the tibial artery in two patients (5.6%). There were no deaths related to these procedures, and the average hospital stay was 3.2 days.

Postoperative management

Most patients were administered third-generation cephalosporins (cefuroxime 50 mg/kg/day in two divided doses) and amikacin (500 mg every 12 hours) for three days. For cases involving prolonged pump time (over 2 hours), extended surgical duration (over 6 hours), emergency surgery, re-exploration, or rising leucocyte or neutrophil count 48 hours post-operation, we used cefoperazone-sulbactam (50 mg/kg/day in two doses) and amikacin (500 mg every 12 hours). In valvular patients, vancomycin was introduced intravenously one hour prior to the surgical incision. We shifted to narrower-spectrum antibiotics as per the corresponding culture and sensitivity findings.

Fast-tracking was defined as extubation within four to eight hours of receiving care in the intensive care unit. We were able to do fast tracking in 82% of valvular surgery patients while 70% of patients with CABG surgery were fast-tracked. Early mobilization within 24 hours was done in 85% of valvular patients and 76% of CABG patients.

ICU discharge policy and length of stay

We prioritized transferring patients from the ICU to step-down units as quickly as feasible to reduce both costs and ICU load. Average ICU stays for patients undergoing open-heart surgery, thoracic surgery, and vascular surgery were 2.2, 2.0, and 1.4 days, respectively, indicating efficient postoperative management and recovery. Longer ICU stays were typically observed in elderly patients post emergency surgery or those experiencing cardiac dysfunction or major complications post-operation.

Complications

We experienced peri-operative complications in 10% of coronary artery bypass graft (CABG) procedures, 12% of valvular and vascular cases, and 7% of thoracic cases. The most severe complications were mediastinal bleeding, necessitating emergency re-exploration, and ventricular tachycardia due to left ventricular dysfunction. Pleural effusion was the most frequent complication during the peri-operative period, affecting 12 patients (9.8%), 10 of whom required a therapeutic pleural tap. Eight patients (6.5%) developed low cardiac output syndrome perioperatively; of these, six needed intra-aortic balloon pump (IABP) insertion along with inotropes. A total of two patients were put on IABP preoperatively while four patients required IABP in the postoperative period. All patients who needed IABP support underwent coronary artery bypass grafting. There was no case of low cardiac output syndrome in adult patients operated on for congenital heart lesions. Complications such as respiratory muscle weakness, bronchospasm, ventilator-associated pneumonia, and hospital-acquired pneumonia were documented in under 2% of patients. Importantly, no vascular complications associated with distal limb perfusion were reported. These complications are detailed in Table 3.

**Table 3:** Perioperative Complications Encountered VT/VF; ventricular tachycardia/ventricular fibrillation, LCOS; low cardiac output syndrome, IABP; intra-aortic balloon pump

Sr. No.	Complications	N / % of Patients	Remarks
1	Bronchospasm	2 / 1.6 %	Associated with weaning failure
2	Malignant Arrhythmia (VT/VF)	4 / 2.3 %	Lignocaine, shock
3	New Onset Atrial Fibrillation	6 / 5 %	All episodes hemodynamically stable, Correction of blood potassium, magnesium, β-blockers
4	Mediastinal Hemorrhage With Hemodynamic Instability	8 / 6.5 %	Four patients required re-exploration
5	Low Cardiac Output Syndrome	8 / 6.5 %	Six patients were managed with IABP
6	Renal Dysfunction	9 / 7.3%	No patient required hemodialysis
7	Surgical Site Infection	10 / 8.2%	Debridement and antibiotics
8	Pleural Effusion-Hemorrhagic	12 / 9.8%	Pleural tap in 10 patients

Perioperative outcomes

Our overall perioperative mortality rate was 4.6%, signifying effective surgical outcomes. Postoperative complications occurred in 8% of patients, which were successfully managed by our medical and surgical teams. Among open-heart surgeries, eight deaths (4.6%) were documented. The causes were: 1) respiratory failure due to COVID-19 pneumonia, 2) postoperative pneumonia leading to respiratory failure, 3) recurring ventricular tachycardia from severe left ventricular dysfunction, 4) respiratory failure related to critical illness myoneuropathy, and 5) a severe transfusion reaction causing cardiovascular collapse. Table 4 provides a detailed overview of the clinical diagnoses and causes of death.

**Table 4:** Mortality Records With Clinical Diagnosis and Cause of Death MR; mitral regurgitation, MS; mitral stenosis, AS; aortic stenosis, MI; myocardial infarction, CHF; congestive heart failure, TVD; triple vessel disease, CPB; cardiopulmonary bypass

Sr No.	Age/sex	Clinical Diagnosis	Operation Done	Cause of Death
1	64/F	RHD, Severe MR, LVEF- 30%	MVR	LV dysfunction
2	61/F	Left Atrial Myxoma	Tumor Removal on CPB	Severe blood transfusion reaction associated with hemolysis (ABO Incompatibility)
3	42/M	CAD, Triple Vessel Disease, With LV Clot, LVEF- 28%	CABG	LV dysfunction, Resistant ventricular tachycardia
4	65/M	CAD- Left Main Disease with Triple vessel Disease, LVEF- 40%	CABG	Resistant VT
5	65/M	RHD- Severe MS, AS	DVR	Infective endocarditits, Abscess rupture
6	75/M	CAD-TVD, LVEF- 40%	CABG	Covid-19 pneumonia, Respiratory failure
7	68/M	CAD- TVD, Acute MI, CHF, IABP in situ	CABG	Pneumonia, Respiratory failure
8	36/F	RHD, Severe MS, LVEF-50%, Severe Muscular Weakness Related to Polio	MVR	Respiratory failure, Critical illness myoneuropathy

## Discussion

Rheumatic heart disease (RHD) continues to be prevalent in India, especially among lower socio-economic groups in rural areas, largely attributed to overcrowding and poor sanitary conditions. The prevalence of RHD ranges from 0.3 to 0.7 per 1000 population in urban areas and 2.2 to 6.6 per 1000 population in rural areas [3]. Underdiagnosis and undertreatment of the disease, particularly in rural areas, contribute to this high prevalence [4]. Moreover, patients with RHD in India often experience high rates of complications and death, as evidenced by data from the World Health Organisation's Global Rheumatic Heart Disease Registry, REMEDY [5].

At the SGRDIMSR facility in Amritsar, clinical diagnoses and subsequent surgical activity reflect the burden of RHD in this region of India. Our most common procedure was valvular replacement for rheumatic heart disease, representing 44% of operations. This experience mirrors that of K.K. Tiwari et al., who performed 85 open-heart surgeries in their first four years, 65% of which were for RHD [6].

The rising prevalence of cardiovascular diseases in India, contributing significantly to the nation's disease burden, is reflected in the high number of patients operated on for coronary artery bypass grafting (CABG) at our facility, similar to trends reported by Kaul et al. [7]. Our surgical team, well-experienced with off-pump procedures, performed most CABG surgeries using this technique. Preoperative screening determined the type of surgery, with 11 cases done on the pump due to preoperative left ventricular dysfunction (<30%). Emergency on-pump conversion occurred in 7.8% of cases due to intractable hemodynamic instability, aligning with Tariq K et al.'s 9% conversion rate [8].

Substance abuse is alarmingly common, particularly peripheral vascular injury due to self-medication with injectable drugs, which accounted for 33% of cases and aligns with trends found by Sharma B et al. [9]. We also noted opioid abuse in 15% of cases, possibly linked to rural cultural practices of consuming opioids (*afeem*) for minor ailments as described by Chavan et al. [10].

The postoperative period brought serious complications. The most common were postoperative low cardiac output (LCOS) (6.8%) and mediastinal hemorrhage (6.8%), similar to previous studies [11-13]. Hospital mortality in the low cardiac output group was significantly higher. Re-exploration for mediastinal bleeding was required in 6.8% of cases, comparable to the 6.0% in the Frojd et al. study [14].

New-onset atrial fibrillation occurred in 5% of open heart surgery cases, a lower incidence as compared to previous studies [15-17]. This may be attributed to our ICU's policy of maintaining blood potassium levels between 4.5 to 5 meq/l and using beta-blocker drugs when possible. Postoperative renal dysfunction was found in 7.3% of cases while the literature reports an incidence of 7-30% [18,19]. This discrepancy may result from the variation in patient characteristics and our practice of restricting diuretic use postoperatively along with goal-directed fluid therapy.

Additionally, we found 12.5% of open heart surgery patients experienced pleural effusion, most of which were hemorrhagic, and 8% required ultrasound-guided therapeutic pleural taps. The incidence of superficial surgical site infection (SSI) was 8.2%, and deep sternal wound infection (DSWI) occurred in 0.6% of cases [20,21]. Ventilator-associated pneumonia was found in 6% of patients, with half of the cases caused by multidrug-resistant Klebsiella pneumonia, similar to findings in a study by Sharma et al. [22]. No complications related to stroke, catheter-related bloodstream infection, or bedsores were recorded in our study.

"Fast Tracking" is a strategy adopted in postoperative care that focuses on expediting patient recovery, particularly following complex surgical procedures like cardiothoracic surgeries. In this context, fast-tracking was operationally defined as the process of extubating patients within four to eight hours after they were admitted into the intensive care unit (ICU).

In our facility, we successfully implemented fast-tracking for a significant proportion of patients. For example, 82% of patients who underwent valvular surgeries were extubated within the specified four to eight-hour window. Similarly, we achieved this rapid recovery timeline in 70% of the patients who had CABG surgery.

In addition to this, another significant aspect of fast-tracking is early mobilization post-surgery. This step is critical in promoting faster recovery and reducing the likelihood of post-surgical complications, such as deep vein thrombosis. Encouragingly, early mobilization within 24 hours was accomplished in 85% of the valvular surgery patients and 76% of the CABG surgery patients.

These figures demonstrate our commitment to improving patient outcomes and enhancing the efficiency of our care delivery. The promising results from our fast-tracking and early mobilization practices reflect the high-quality care provided at our facility, emphasizing our capacity to handle complex surgical procedures in a tier-2 city setting effectively.

In our study, the overall mortality rate observed was 4.6%, a figure that aligns closely with the 5.7% mortality rate noted by Prachi Kar et al. in their research [23]. This comparison, which is on par with previous studies, underscores the success of our facility in maintaining a comparable standard of care within a tier-2 city setting.

Examining the operative mortalities more closely, the majority were linked to cardiac complications post-CABG and mitral valve surgeries. A significant percentage of these mortalities, 75%, occurred within the critical early postoperative period, specifically within the first three days post-surgery. The remaining 25% of deaths happened within the subsequent two days, emphasizing the vulnerability of this initial postoperative period.

Upon investigating the cause of these mortalities, we found that ventricular dysfunction and associated arrhythmias were the most common causes, accounting for 50% of the cases. This highlights the significant role that cardiac complications play in operative mortalities following cardiothoracic surgeries. Additionally, respiratory failure was a significant cause of death, leading to 37% of mortalities.

The findings underscore the critical importance of vigilant postoperative monitoring, particularly within the first few days following surgery. These are key areas for future improvement and interventions to further optimize patient outcomes and reduce postoperative mortality rates. This detailed analysis demonstrates the complexity of addressing cardiovascular health in this region, shedding light on the multiple dimensions of surgical and postoperative care, as well as socio-cultural influences that must be considered in our ongoing efforts to improve cardiothoracic and vascular surgery outcomes in tier-2 cities in India.

Challenges faced

Creating a successful cardiothoracic and vascular surgery (CTVS) unit faced initial challenges due to limited resources, staffing challenges, and staffing issues. In 2016, the European Association for Cardio-Thoracic Surgery (EACTS) published a clinical statement outlining modern cardiac surgical unit layout suggestions for healthcare professionals, chief executive officers, and medical directors to design successful programs for adult cardiothoracic conditions [24].

Further, as a new medical facility, we faced the difficult task of building trust with potential patients. This was particularly challenging in the CTVS domain due to the substantial risks associated with the procedures we perform. Alongside this, raising awareness about our newly established facility and its services was a necessity that demanded significant effort and resources.

Another hurdle was the limited patient volume we faced in the initial phase. This could be attributed to the relatively smaller population base, which limited the number of individuals requiring cardiothoracic and vascular surgery. The low patient volume threatened the unit's financial sustainability. Moreover, we initially had fewer robust patient referrals, potentially leading to even fewer patients visiting our facility. Lastly, establishing processes for ongoing quality assurance, including obtaining accreditation from relevant professional organizations like the National Accreditation Board for Hospitals (NABH), represented another significant obstacle in our journey.

Limitations of the study

This study, focusing on the early stages of a cardiothoracic surgery facility in a tier-2 city in India, has key limitations. First, its two-year timeframe may not adequately depict long-term outcomes or sustainability. Second, as the findings derive from a single center, they might not reflect experiences in other settings. The limited sample size also raises questions about the statistical power and generalizability of results. The outcomes might have been influenced by the facility's newness, limited resources, and the skill level of the personnel. Furthermore, without a control group, it's difficult to compare the facility's relative success. The study's lack of long-term patient outcome tracking, quality of life measurements post-discharge, and economic evaluation also limit its scope and applicability, leaving questions about cost-effectiveness unanswered. Future research should address these limitations.

Evaluation and future recommendations

The outcomes of the center were continuously evaluated with feedback from patients, healthcare professionals, and administrative staff. This feedback was then used to inform changes and improvements in the center's operations. Based on the experience gained, several recommendations can be made for establishing similar healthcare facilities in underserved regions. These recommendations are summarized in Table 5.

**Table 5:** Recommendations for Establishing a Similar Healthcare Facility

Recommendation for Setting Up a Successful CTVS Unit	
1	Strategic collaborations with established institutions for staff training and knowledge sharing.
2	The robust recruitment process for hiring qualified staff.
3	Continued professional development programs.
4	Sustained community engagement and awareness initiatives to increase patient acceptability for cardiac surgery.
5	Emphasizing quality improvement and patient safety measures to improve patient outcomes.
6	Establishing a better patient referral network to increase patient volume.
7	Establishing protocols regarding substance abuse and hepatitis seropositivity.
8	A routine audit of patient morbidity and mortality for quality improvement.

## Conclusions

The establishment of a cardiothoracic surgery facility in a Tier-2 city in India has proven to be a feasible and successful venture over the initial two-year period, offering specialized cardiac care to the local populace. Even in the face of various challenges, the facility managed to achieve positive perioperative outcomes, effectively handle complications, and provide critical surgical services. These findings provide crucial insights and lessons for future efforts intending to establish similar healthcare facilities in underserved regions, thereby promoting improved access to specialized care and reducing health disparities.
